# Microablative fractional radiofrequency as a therapeutic option for vulvar lichen sclerosus: a pilot study

**DOI:** 10.6061/clinics/2021/e2567

**Published:** 2021-03-19

**Authors:** Márcia Farina Kamilos, Lana Maria Aguiar, Valéria Holmo Batista, Cristiane Lima Roa, Fernando Nalesso Aguiar, José Maria Soares, Edmund Chada Baracat

**Affiliations:** I Disciplina de Ginecologia, Departamento de Obstetricia e Ginecologia, Hospital das Clinicas HCFMUSP, Faculdade de Medicina, Universidade de Sao Paulo, Sao Paulo, SP, BR.; II Patologia, Instituto do Cancer do Estado de Sao Paulo (ICESP), Hospital das Clinicas HCFMUSP, Faculdade de Medicina, Universidade de Sao Paulo, Sao Paulo, SP, BR.

**Keywords:** Vulvar Lichen Sclerosus, Vulvar Itching, Atrophy, Radiofrequency Treatment, Corticosteroids

## Abstract

**OBJECTIVES:**

To assess the clinical response to and the histomorphometric effects of microablative fractional radiofrequency (MFR) in women with symptomatic vulvar lichen sclerosus (VLS).

**METHODS:**

This was a pilot study on the use of MFR for the treatment of VLS. Upon recruitment and at each treatment session, all participants were examined and each of their symptoms were rated on a visual analog scale. After the procedure, the participants completed a satisfaction questionnaire. We compared the morphometric findings of vulvar biopsies performed at enrollment and after the last treatment session. The participants were divided into three groups according to previous treatment with corticosteroids: G1, no previous treatment; G2, treated for up to 5 years; and G3, treated for >5 years.

**RESULTS:**

This study included 26 women. After two to three sessions, most participants in all groups became either “asymptomatic” or “much better” than before treatment and were “very satisfied” or “satisfied” with the intervention. Pruritus and burning sensation were the most frequently reported symptoms. Nearly 40% of the participants in all groups reported complete remission of symptoms. The improvement was rated as moderate or higher by 80%, 76%, and 66% of the women in groups 1, 2, and 3, respectively. The improvement of symptoms persisted for 11 months (range, 7-16 months), on average, after the treatment. Type III collagen concentration significantly increased and was associated with important symptom improvement. Tissue trophism and vascularization also increased but did not reach statistical significance, probably because of the small number of cases.

**CONCLUSIONS:**

MFR may be an effective and safe treatment for symptomatic VLS.

## INTRODUCTION

Lichen sclerosus (LS) is a chronic progressive dermatosis that can affect different areas of the skin; however, in 80-90% of patients, it occurs in the anogenital region (1). LS affects mostly women (five women to one man), especially during the postmenopausal period (2-6). Typically, vulvar LS (VLS) presents as atrophic, ivory white areas in a “number 8” pattern (vulvar, perineal, and perianal involvement), with depigmentation or hyperpigmentation, ecchymoses, minor labia resorption, introital narrowing, and vulvar architecture distortion, which can progress to minor labia coalescence and introital stenosis. The main symptoms are pruritus, especially at night, followed by pelvic pain, dyspareunia, and burning sensation. These symptoms may affect sexual activity and contribute to the high prevalence of sexual dysfunction in these patients (7-12).

Three important features are prominent in women with VLS: intense pruritus, which is more frequent after menopause, an increased risk for differentiated vulvar intraepithelial neoplasia, and progression to keratinized vulvar squamous cell carcinoma in 4-6.7% of the patients (4). Strict patient adherence to ideal treatment, along with appropriate follow-up, can modify the course of the disease and reduce the risk of malignant transformation. However, some women with recalcitrant VLS find it difficult to adhere to prolonged treatment with corticosteroids or estrogen (7,10).

At present, there is no cure for VLS. The first-line treatment is the topical use of highly potent corticosteroids to avoid fibrous scars and potential progression to more severe forms of the disease. There is some evidence that women with a recent diagnosis of VLS respond better to treatment (7,13-15). However, corticosteroids should be used in the lowest amounts and frequency, and for the shortest period needed to promote clinical improvement, owing to their adverse effects (7-13). In addition, corticosteroids inhibit fibroblast proliferation and reduce collagen production, which are harmful for the vulva. The most frequent adverse effect of topical corticosteroids is local cutaneous atrophy. Other local adverse effects include skin fragility, hypopigmentation, burning sensation, drying, telangiectasias, and fungal superinfections (16). However, patient adherence to this treatment has been low for a long time. Therefore, alternative therapeutic options have been evaluated, such as fractioned CO_2_ laser, radiofrequency, photodynamic therapy, and high-intensity focused ultrasound (7,15-18).

Laser and fractioned radiofrequency are used to improve general skin and mucosal trophism, especially in the vagina and vulvar vestibule. Several researchers have reported that these interventions produce clinical improvement, as well as promising results in neocollagenesis and neoelastinogenesis assessed using histopathology, electron microscopy, and immunohistochemistry (8,18-24). According to Condi et al. (25), dermal collagen can be assessed by measuring collagen I and II fractions, and neocollagenesis can be evaluated using picrosirius staining with polarized microscopy to highlight fiber width and orientation. Morphometric techniques can be used to quantify these changes. Junqueira et al. (26) developed a method with polarized picrosirius to identify collagen I, II, and III on routine histological slides, which can be used to study the distribution of different types of interstitial collagen. This method has also been used by other researchers (27).

Microablative fractional radiofrequency (MFR) has been used to treat several dermatological and gynecological conditions, but not in patients with VLS. We assessed the potential benefits of MFR on epithelial trophism and symptom improvement, as well as the duration of its effect. Regardless of previous corticosteroid treatment, we hypothesized that MFR sessions would improve clinical symptoms and local atrophy and promote histomorphometric changes, and that these effects would be prolonged, lasting at least 6 months after the last session.

## MATERIALS AND METHODS

### Study design and participants

This pilot case series involved 26 postmenopausal women with symptomatic, histologically confirmed VLS managed in a single specialized benign vulvar pathology clinic (Hospital das Clínicas da Faculdade de Medicina de São Paulo [HC-FMUSP]) located in São Paulo, Brazil. We divided the participants into three groups according to previous use of topical corticosteroids: G1, no previous use; G2, up to 5 years of use; G3, >5 years of use. All participants were recruited between June 4, 2018, and October 27, 2019. We excluded women with any of the following: previous treatment for intraepithelial neoplasia, carcinoma, or local radiotherapy. The participants were instructed not to use corticosteroids for 3 months before study enrollment. They were also asked to avoid using topical corticosteroids throughout the MFR treatment period. They were allowed to use an emollient, if necessary.

### Intervention

All participants underwent one to three MFR sessions at 30-120-day intervals using a Wavetronic 6000HF-FRAXX machine (Loktal Medical Electronics, São Paulo, Brazil). The equipment characteristics were as follows: electromagnetic generator with 4-MHz oscillating frequency and electronic energy fractioning circuit connected to a skin electrode with 64 microneedles (0.2-mm width × 0.8-mm length) disposed in eight rows (eight needles per row) that produce microablations at 1-mm intervals. Each needle receives either 225 or 338 mJ when the device is set at low or medium energy, respectively.

Before each session, and 60-180 days after the last session, we performed a physical examination of the participants and photographed the area of interest. Moreover, the participants were asked to assess the intensity of each symptom using a visual analog scale (VAS). At the end of each session, the participants were asked to complete a satisfaction questionnaire using a Likert scale.

We conducted histomorphometric analyses of vulvar biopsy specimens obtained before the first and after the last session in 11 participants. The specimens were sectioned, fixed in 10% neutral buffered formalin, and embedded in paraffin. Slides were stained with hematoxylin and eosin and picrosirius, and examined using optical microscopy (Nikon^®^ Eclipse E200LED). The following histological parameters were assessed using hematoxylin and eosin staining: epithelial thickness (using a microscope ruler) and type (atrophic or trophic), stromal cellularity (mild, moderate, or marked), stromal vascularization (slight, moderate, or intense), and inflammatory infiltration (slight, moderate, or intense). We used a polarized lens coupled to the microscope to assess the percentage of type I or III collagen fibers in the stroma.

We used the 45-W equipment protocol and selected the low-energy setting for women with a thin atrophic vulvar epithelium and the medium-energy setting for those with a thicker epithelium (for a deeper thermal effect on the dermis). Each session lasted an average of 15-20 min. Mild edema and hyperemia occurred in the treated skin areas, with spontaneous resolution within 1-3 h. Signs of microablation completely disappeared from the vestibular mucosa within 3 days and from the skin within 5-7 days. After each session, we instructed the participants to use 5% dexpanthenol cream and/or cold saline compresses two or three times per day for 2-5 days, and to abstain from sexual intercourse for the next 7 days.

### Questionnaires

- VAS: This scale was used to assess the intensity of each symptom at baseline, at each MFR session, and during the follow-up period. The physician asked the participant to point to the number on the scale that best represented the intensity of her symptom/pain (0, complete absence of symptoms; 10, maximum symptom intensity).
- Current *versus* pretreatment status scale: The participants were asked to select the words that best described how they currently felt, compared to their pretreatment status, on a five-point Likert scale (completely asymptomatic, much better, somewhat better, unchanged, or worse).
- Post-procedure satisfaction questionnaire: At the end of each MFR session, the participants were invited to grade their satisfaction with the intervention on a five-point Likert scale (very satisfied, satisfied, unsure, unsatisfied, or very unsatisfied).

### Statistical aspects

The participants’ sociodemographic characteristics are presented as means and standard deviations for continuous variables (*e.g.*, age), and as frequencies and percentages for categorical variables (*e.g.*, hypertension). We used the Wilcoxon nonparametric test to assess differences among groups in the mean scores before and after treatment. We used Fisher’s exact test to assess the differences in percentages among the groups. We used a mixed-model analysis of variance, which considers repeated measures per participant, to assess changes in scale scores between sessions in the groups. We used Pearson’s linear correlation coefficient to assess the correlation between symptom duration (in years) and improvement (absolute and percentage estimates). This test measures the degree of linear association between two variables, and the correlation between symptom improvement and duration. Statistical significance was set at *p*<0.05. Stata/SE 10.0 statistical software (Stata Corporation, College Station, TX, USA) was used for all statistical analyses.

### Ethics statement

The study was approved by the HC-FMUSP ethics committee (CAAE 00449418.0.0000.0068) and conducted according to resolution 196/96 of the National Health Council, which regulates human research in the country. All participants provided written informed consent at enrollment.

## RESULTS

We included 26 women: 5 (19.2%) in G1, 5 (19.2%) in G2, and 16 (61.5%) in G3. The mean age was 61.9 years (±9.1 years, standard deviation), ranging from 44 to 82 years. There was no statistically significant difference in the mean ages of the participants in G1, G2, and G3 (61.6, 56.8, and 63.6 years, respectively, *p*=0.465). As expected, there was a significant difference among the three groups in the mean duration of symptoms (1.4, 3.4, and 11.7 years in G1, G2, and G3, respectively; *p*<0.0001).

The most frequent comorbidity was hypertension, with significant differences among groups (100%, 20%, and 50% in G1, G2, and G3, respectively; *p*=0.047). All other comorbidities had a low prevalence, affecting <20% of the women in each group. Each participant had zero to four comorbidities, without significant differences among the groups.

We excluded one participant because of an intense herpes episode after the first MFR session. She was using acyclovir at the time and had a history of similar episodes in the past. Two other women with a history of genital herpes did not have any new episodes during the study or follow-up period.

**Figure 1:**
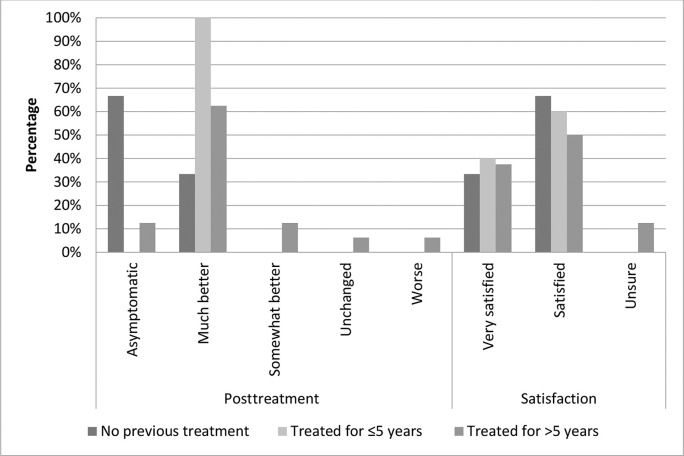
Clinical data of the participants after treatment compared to their baseline status, and the participants’ satisfaction with MFR. Group 1, no previous corticosteroid treatment; group 2, treated for up to 5 years; group 3, treated for >5 years.

At study enrollment, pruritus was the most frequently reported symptom by all participants, without significant differences among the groups (*p*=0.631). To assess symptom improvement during treatment, we compared the VAS intensity scores of each participant’s main symptom at baseline *versus* after the last session (for most participants this was the third session, for some, it was the first or second session). Most participants in all groups reported that they felt “much better” or “asymptomatic” (G1: 100%, G2: 100%, and G3: 75%) after MFR. Four patients in G3 (25%) reported that they felt “somewhat better” or “worse” after the last session (Figure 1); however, the difference was not statistically significant (*p*=0.453). The vast majority of participants reported feeling “very satisfied” or “satisfied” (100%, 100%, and 87.5% in G1, G2, and G3, respectively). Two patients in G3 (12.5%) reported that they felt “unsure”; however, this was not statistically significant (*p*=1.00) (Figure 1).

The mean status change (complete, intense, moderate, slight, or none) was somewhat higher in G1 than in the other groups; however, the difference was not statistically significant (*p*=0.747). Nearly 40% of the women in each group showed “complete improvement.” Moderate or higher improvement (including complete improvement) occurred in 80%, 76%, and 66% of women in G1, G2, and G3, respectively. Symptom improvement persisted for an average of 11 months (range, 7-16 months) after the last session.

**Figure 2:**
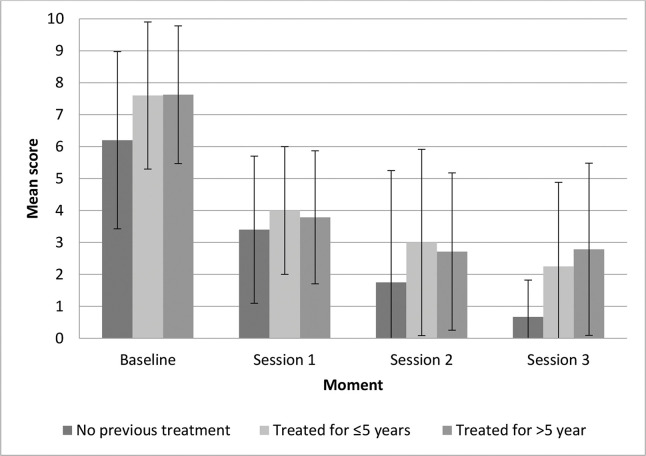
Symptom intensity scores during MFR treatment (mean and standard deviation).

The participants in G1 had a steady decrease in the mean symptom intensity scores throughout the treatment (Figure 2). In G2 and G3, the decrease was apparently more striking following the first session than afterwards. However, there were no significant differences in symptom intensity improvement among the three groups (*p*=0.975). Symptom intensity improvement was lower in women with longer disease duration, although we found no significant correlation between symptom intensity improvement and symptom duration.

**Figure 3:**
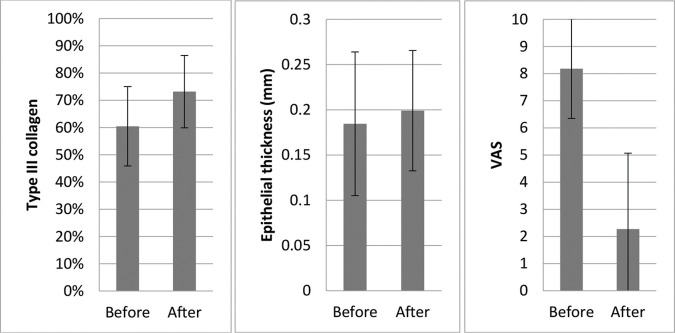
Changes in type III collagen concentration, epithelial thickness, and symptom intensity scores before and after MFR treatment.

Eleven patients had histology data before and after treatment (Figure 3):

1. Collagen: Overall, there was a significant increase in the concentration of type III collagen (from 60.5% to 73.2%) and a proportional decrease in type I collagen (*p*=0.008).
2. Changes in symptom intensity (VAS) in the patients: Overall, there was a large and statistically significant reduction in the mean symptom intensity scores (from 8.2 to 2.3, *p*=0.002), which corresponded to an average improvement of 72.6%.
3. Epithelial thickness: There was a slight increase in mean epithelial thickness (from 0.185 to 0.199 mm); however, this was not statistically significant (*p*=0.711).
4. Trophism: None of the women had atrophic epithelium after the last MFR session, compared to 36.4% at baseline. This was not statistically significant, probably because of the small sample size.
5. Stromal cellularity: There was a small change in this parameter. Two patients had moderate to mild cellularity (72.7-90.9%); however, this was not statistically significant (*p*=0.157).
6. Vascularization: There was little change in this parameter. Two patients showed a change from intense to slight and moderate vascularization, whereas two other patients showed a change from slight and moderate to intense vascularization. These changes were not statistically significant (*p*=1.000).
7. Inflammatory infiltration: After the intervention, five patients had less intense inflammatory infiltration and one patient had more intense inflammatory infiltration. These changes were not statistically significant (*p*=0.262).

## DISCUSSION

There was a satisfactory and sustained improvement in VLS symptoms in almost all women with a more recent onset of disease, especially in those who had not been previously treated with corticosteroids. These findings support the belief that earlier treatment is associated with better results in women with VLS (5). Despite the small sample size, the results suggest the favorable outcome of MFR. It was difficult to recruit women with VLS without previous local corticosteroid treatment, probably owing to delays in patient referral from primary healthcare settings or in the diagnosis of the disease.

Women without previous treatment (G1) had sustained symptom severity improvement in all MFR sessions, whereas women with previous treatment (G2 and G3) showed a more intense improvement after the first session, declining thereafter. It is possible that women with long-standing VLS and partial improvement could benefit from more MFR sessions. The adverse effects of MFR were minimal and transient, consisting mainly of local hyperemia and burning sensation, sometimes associated with urinary urgency, which spontaneously resolved within a few hours of the procedure. The sessions were well tolerated, and there were no permanent complications in any woman throughout the study. For women with genital herpes, we recommend the use of acyclovir or famciclovir for 5-7 days, starting on the day before the scheduled procedure.

**Figure 4:**
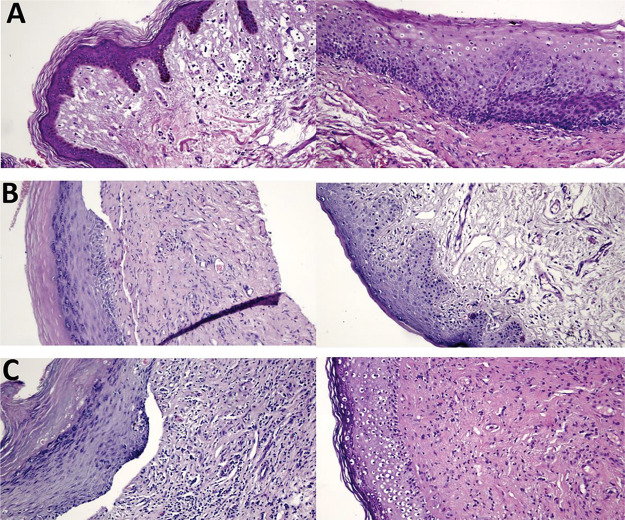
Hematoxylin and eosin-stained histological slides. Pretreatment (left) and posttreatment (right) with MFR. **A**. Group 1 patient, age 62 years, 8 months since diagnosis: 100% improvement in pruritus, asymptomatic and satisfied. **B.** Group 2 patient, age 72 years, 4 years since diagnosis: 100% improvement in pruritus, persistent improvement at 12 months after treatment, much better and very satisfied. **C**. Group 3 patient, age 82 years, 7 years since diagnosis: 100% improvement in pruritus, much better and very satisfied.

Histological studies have shown that women with VLS have dermal sclerosis and inflammation (11). Some women in our study had reduced inflammatory infiltration and improved vascularization; however, these changes did not reach statistical significance. In addition, all women with atrophic epithelium at baseline had trophic epithelium after treatment. Three of our participants had a clear reduction in hyperkeratosis, epidermal maturation, and reduction in inflammatory infiltration after treatment (Figure 4).

We also observed an increase in the proportion of type III to type I collagen after treatment. This suggests that MFR may improve “elasticity” or “plasticity” by increasing the number of thinner type III collagen fibers. This histological finding was associated with a significant improvement in the clinical symptoms (Figure 3).

Several factors may have influenced our histological analyses. Despite our instructions, a few participants admitted that they had used local corticosteroids for short periods during the study. It is also possible that we may not have selected the most representative areas for the biopsies in all participants.

Although several studies have compared techniques with the same objective (28-30), to our knowledge, there are no comparative studies among different types of radiofrequency equipment, including differences in energy settings, modes (ablative, microablative, or nonablative), needle types and sizes, and whether the needles perforate the tissue to produce thermal stimuli.

**Figure 5:**
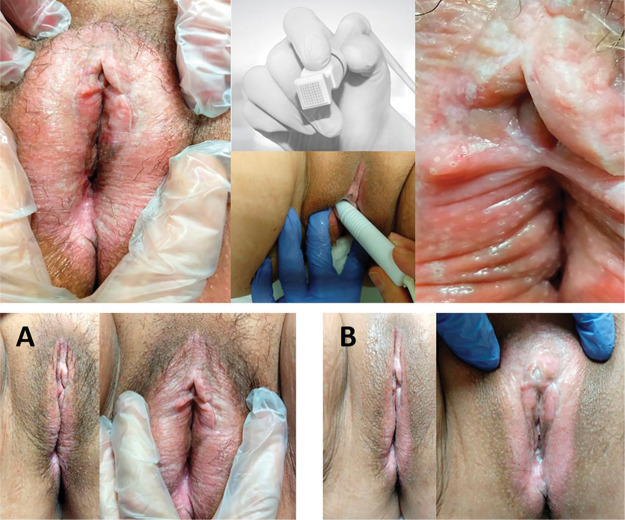
A 65 year-old patient with VLS diagnosed 22 years prior, with pretreatment and posttreatment pruritus intensity VAS scores of 10 and 5, respectively. The patient reported feeling “somewhat better” but “satisfied” after MFR treatment. Upper photos: microablations in MFR sessions and the fractioned electrode. Lower photos: vulvar appearance before treatment (A), and improved skin texture and color after treatment (B).

The MFR equipment used in this study has a fractioning system that produces areas of denatured collagen adjacent to areas of normal tissue next to the vaporized spots. The equipment also has a random pulse that produces thermal microdamage at a measurable depth in the reticular dermis, without significant lateral thermal effects, to stimulate tissue regeneration without producing scars (Figure 5). The needles gently touch the tissue, without perforating it, to transmit an electromagnetic current. Biopsies in vertical ablative perforations in patients treated with MFR have shown that the thermal, nonablative effect reaches 0.1 mm in depth with minimal lateral thermal effect. Horizontally, there is a thermal effect on the dermis just below the microablation, with completely preserved tissue at 1-mm intervals between perforations (24).

Fractional CO_2_ lasers produce effects similar to those of MFR. This type of laser has been successfully used to treat vaginal atrophy and VLS (15). A cohort study involving 27 women with symptomatic LS reported that 89% had complete regression of pruritus and pain after three or four fractioned CO_2_ laser sessions 4-6 weeks apart (14). In addition, double-blind, randomized, placebo-controlled trials comparing fractional CO_2_ laser with topical corticosteroid treatment for women with VLS are missing in the literature (31).

The first pilot study on vaginal and vestibular MFR for genitourinary menopausal syndrome reported that the intervention was effective on atrophy symptoms, was well tolerated, and was associated with a rapid recovery; however, the study included only a few women (21). More robust studies have confirmed that MFR can be beneficial in the treatment of patients with genitourinary disorders (22,23). Sarmento et al. (22) conducted a randomized trial on the effects of MFR on vaginal health, microbiota, and cellularity in postmenopausal women. The preliminary results indicated that MFR considerably improved the vaginal microenvironment, similar to that expected in women with adequate estrogen concentrations. According to the authors, these findings suggest that radiofrequency can improve the vaginal symptoms of genitourinary menopausal syndrome.

Owing to high recurrence rates in patients who stop using drugs after symptom remission, chronic inflammatory dermatological disorders frequently require the continuous use of suppressive medications (5). Therefore, maintenance therapy is recommended even after the patients become asymptomatic (10). Women with VLS have a considerable risk of scarring, loss of architecture, and vulvar dysfunction (5). Cooper et al. (9) analyzed 327 women with delayed VLS diagnosis, and reported fewer scars in those diagnosed within the first 2 years of symptom onset.

The benefits of MFR observed in our participants included improved trophism of the vulvar skin and mucosa. Intravaginal MFR can extend these benefits to the vaginal wall in patients with VLS associated with vaginal atrophy.

A strong point of our study was the inclusion of participants without previous treatment, which can potentially better demonstrate the effects of the intervention. Additionally, we used fractioned radiofrequency, an innovative, easy-to-use intervention with a rapid recovery period. In this study, MFR produced a relevant and persistent improvement in symptoms, which was associated with patient satisfaction. These findings suggest that MFR can be considered as the first therapeutic option or can be used to complement medical treatments for VLS. A study limitation was that we did not compare MFR with other types of thermal energy treatment and the small number of included patients (pilot study).

Our findings open new therapeutic possibilities for the prolonged control of VLS symptoms. Biannual or annual MFR maintenance sessions can help improve patient adherence to long-term follow-up. Although VLS is a chronic disease, there is always hope for complete disease remission.

Further studies are needed to compare MFR with other VLS treatments and to assess the long-term effects of the intervention.

## CONCLUSION

MFR may be effective in the long-term relief of anogenital LS symptoms, especially pruritus, burning sensation, dryness, and dyspareunia. Further studies are needed to confirm the clinical findings of this pilot study.

## AUTHOR CONTRIBUTIONS

Kamilos MF, Aguiar LM, Batista VH, Roa CL, Aguiar FN, Soares-Júnior JM and Baracat EC provided substantial contributions to the conception, design, data collection, analyses and interpretation, manuscript drafting or critical review relevant to intellectual content. All of the authors approved the final version of the manuscript.
